# Performance optimization of InSe-FETs using high-k dielectric materials for analog/RF applications

**DOI:** 10.1038/s41598-025-21242-9

**Published:** 2026-03-10

**Authors:** Md Akram Ahmad, Muzaffar Imam, Bhubon Chandra Mech, V. Bharath Sreenivasulu

**Affiliations:** 1 Department of Electrical, Electronics and Communication Engineering, Galgotias University, Greater Noida, 203201 India; 2 Department of Computer Science and Engineering, Jamia Hamdard University, New Delhi, New Delhi, 110062 India; 3 Department of Electronics Engineering, Defence Institute of Advanced Technology, Pune, 411025 India; 4 Department of Electronics and Communication Engineering, Manipal Institute of Technology Bengaluru, Manipal Academy of Higher Education, Manipal, India

**Keywords:** 2D material, Transition metal dichalcogenide (TMD), Field-effect-transistor (FET), High-k material, Analog/RF, Non-equilibrium Green’s function (NEGF), Materials for devices, Nanoscale materials

## Abstract

This study investigates the performance of Indium Selenide (InSe)-based field-effect transistors (FETs) incorporating high-k dielectric materials, using atomistic simulations based on the non-equilibrium Green’s function formalism. Initially, the effect of high-k dielectrics on the ON/OFF current ratio (*I*_*ON*_/*I*_*OFF*_) is analyzed to assess their suitability for digital applications. Subsequently, key analog and radio-frequency (RF) performance metrics, such as intrinsic gain (*A*_*V*_), transconductance generation factor (TGF), and cut-off frequency (*f*_*T*_), are examined. The results demonstrate notable enhancements: transconductance (*g*_*m*_) increases by 98.3%, TGF by 73.7%, and *A*_*V*_ by 72.66%. However, a 9.85% reduction in *f*_*T*_ is observed, indicating a trade-off between gain and speed. These findings highlight the potential of high-k dielectric engineering in optimizing InSe-based FETs for next-generation analog and RF circuits.

**Supplementary Information:**

The online version contains supplementary material available at 10.1038/s41598-025-21242-9.

## Introduction

Two-dimensional van der Waals (2D vdW) materials possess a characteristic layered structure in which strong in-plane covalent bonds hold atoms within a layer, while interlayer interactions are governed by relatively weak van der Waals forces. These weak interlayer forces facilitate the mechanical exfoliation and transfer of individual layers onto various substrates without significant structural degradation. Prominent examples of 2D vdW materials include graphene and transition metal dichalcogenides (TMDs) such as MoS_2_, MoSe_2_, MoTe_2_, WS_2_, and WSe_2_. Due to their excellent physical and electrical properties, these materials are considered promising candidates for post-silicon electronics, particularly in the development of flexible and transparent electronic devices^1^.

While graphene is renowned for its high carrier mobility, its zero bandgap limits its use in digital electronics. In contrast, TMDs offer sizable bandgaps and favorable electronic properties, making them suitable for low-power nano-electronic applications. For example, monolayer MoS_2_ exhibits a bandgap in the 1–2 eV range along with relatively high effective masses, which help mitigate source-to-drain leakage currents. Although challenges such as controlled doping and achieving low-resistance contacts remain^2^, substantial advancements have been made, and no critical bottlenecks currently hinder their continued development.

Following the progress in TMDs, attention has shifted to another class of layered materials known as group-III monochalcogenides, including GaS, GaSe, GaTe, and Indium Selenide (InSe)^3^. Among them, InSe has demonstrated excellent electronic and optoelectronic characteristics in few-layer configurations. It exhibits high carrier mobility at room temperature and strong light-matter interactions^4–7^, making it a promising material for flexible photonic and photovoltaic applications^8,9^. Importantly, the optical and electronic properties of InSe can be tailored by adjusting the number of layers, allowing for transitions from direct to indirect bandgaps and corresponding changes in bandgap energy, crucial for optimizing optoelectronic device performance^9^.

Several studies have reported the development of photodetectors using InSe or InSe–graphene heterostructures, showing enhanced responsivity, broader spectral detection, and efficient photoconversion^10,11^. In addition, research on InSe-based field-effect transistors (FETs) has explored the influence of temperature, substrate interactions, and remote phonon scattering (particularly from high-k dielectrics) on carrier mobility^12,13^. Simulation-based studies suggest that monolayer InSe-FETs exhibit excellent performance as n-type devices when the channel length exceeds 7.2 nm^14,15^. These devices offer ON-current (*I*_*ON*_), low OFF-current (*I*_*OFF*_), and strong electrostatic gate control^14^. Recent theoretical works have also investigated carrier transport in few-layer InSe-FETs^16^, and experimental demonstrations of bilayer InSe-FETs have achieved impressive room-temperature mobilities exceeding 10^3^ cm^2^V^−1^ s^−1^, reinforcing their potential for next-generation high-performance electronic devices^17,18^.

Despite growing interest in InSe for digital and optoelectronic applications, its potential in analog and radio-frequency (RF) domains remains relatively unexplored. This study aims to address this research gap by evaluating the analog and RF performance of monolayer n-type InSe-FETs using the non-equilibrium Green’s function (NEGF) formalism. It investigates the impact of high-k dielectric engineering on performance metrics critical to analog/RF circuits, such as the gain frequency product (GFP), transfer frequency product (TFP), and gain transfer frequency product (GTFP). These metrics are essential for high-speed, energy-efficient circuit design. The findings offer valuable guidance for the future development of InSe-based analog and RF electronic devices.

## Device structure and simulation methodology

**Fig. 1:**
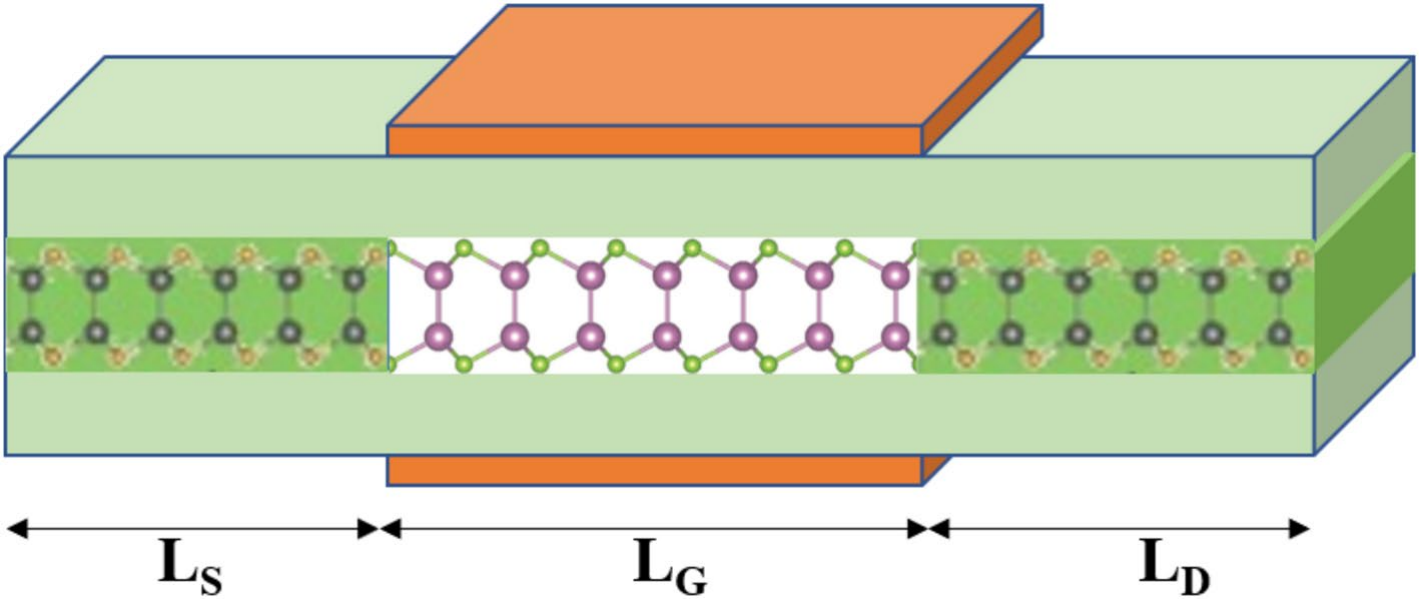
The Schematic view of the simulated InSe-FET.

The schematic view of the simulated InSe-FET is depicted in Fig. 1. The channel and source/drain regions have equal lengths. The source and drain regions are doped with n-type dopants, whereas the channel region remains intrinsic. Monolayer InSe exhibits a honeycomb lattice structure consisting of two selenium (Se) atoms and two indium (In) atoms positioned at non-equivalent sites.

This arrangement forms a four-atom stack in the sequence Se–In–In–Se, with In–In and In–Se bond lengths of 2.79 Å and 2.65 Å, respectively. Aluminium, with a work function of 4.1 eV, is used as the gate material. A one-dimensional armchair-edged InSe nanoribbon structure is employed in the simulation, with a width to suppress the influence of edge states on transport characteristics. The device simulation parameters are listed in Table 1. The performance of the InSe-FET is assessed based on the International Roadmap for Devices and Systems (IRDS) 2021 projections for CMOS technology^19^.

**Table 1:** Parameters of the InSe-FET structure.

Symbols	Definition	Nominal values
L_G_	Gate length	7.5 nm
L_S_	Source length	7.5 nm
L_D_	Drain length	7.5 nm
t_ox_	Oxide thickness	1 nm
N_D_	Source and Drain doping	3.6 × 10^13^ cm^−2^
E_G_	Gap Energy	1.5 eV
m_h_	Effective mass for holes	1.8 *m*_0_
m_e_	Effective mass for electrons	0.16 *m*_0_
V_DS_	Drain-source voltage	0.5 V

To investigate the linearity behavior of InSe-FET, we employ two-dimensional atomistic simulations based on the Non-Equilibrium Green’s Function (NEGF) formalism using the NanoTCAD ViDES framework^20^. This quantum transport methodology captures essential nanoscale phenomena such as ballistic conduction. The electronic structure is described using a *p*_*z*_-orbital-based tight-binding Hamiltonian, incorporating a nearest-neighbour hopping parameter (*t*) of *t* =  − 2.7 eV. The model uses a two-band formulation, where energy states near the conduction and valence band edges are defined, and their separation corresponds to the material’s bandgap.

The two-band Hamiltonian is defined as^21^:

1 $$H\left(k\right)=\left[\begin{array}{cc}{E}_{B}& tf(k)\\ {tf(k)}^{*}& {E}_{A}\end{array}\right]$$

here energy states near the conduction and valence band edges are denoted as ${E}_{B}$ and ${E}_{A}$ respectively, whereas *f*(*k*) is calculated as^22^:

2 $$f\left(k\right)={(1+e}^{-j{k}_{y}{a}_{1}}+{e}^{-(j{k}_{y}{a}_{1}+j{k}_{y}{a}_{2})})$$

here *a*_1_ and *a*_2_ represent primitive lattice vectors of the material.

Once the Hamiltonian matrix is formulated, the next step involves computing the Green’s function as given by^23,24^:

3 $$G\left( E \right) = \left[ {EI - H - \sum_{S} - \sum_{D} } \right]^{ - 1}$$

To account for the source and drain contacts’ impact, corresponding self-energy matrices ($\sum_{S}$ and $\sum_{D}$) are introduced to the Hamiltonian matrix. These matrices are incorporated into the Hamiltonian to capture the open-boundary conditions at the device terminals. The simulation assumes ballistic transport and considers an armchair-edged InSe nanoribbon structure. The chemical potentials of the source and drain reservoirs are assumed to be in equilibrium with the Fermi level of the InSe channel. Due to the absence of fully confined states in the channel, carrier statistics are determined through electron and hole concentration equations^20^:

4 $$n(\vec{r}) = 2\int_{{E_{i} }}^{ + \infty } {dE\left[ {|\psi_{S} (E,\vec{r})|^{2} f(E - E_{{F_{S} }} ) + |\psi_{D} (E,\vec{r})|^{2} f(E - E_{{F_{D} }} )} \right]}$$

5 $$p(\vec{r}) = 2\int_{{E_{i} }}^{ + \infty } {dE\left\{ {|\psi_{S} (E,\vec{r})|^{2} \left[ {1 - f(E - E_{{F_{S} }} )} \right] + |\psi_{D} (E,\vec{r})|^{2} \left[ {1 - f(E - E_{{F_{D} }} )} \right]} \right\}}$$

These carrier densities are subsequently input into a self-consistent Schrödinger–Poisson solver^25^, which utilizes the Newton–Raphson iterative method^26^ to compute the electrostatic potential, subject to specified convergence criteria. Once convergence is achieved, drain current (I_D_) is calculated using the Landauer formalism^23,24^:

6 $$I_{D} = \frac{2q}{h}\int_{ - \infty }^{ + \infty } {dE\left\{ {Tr\left[ {\left( {\Sigma_{S} - \Sigma_{S}^{\dag } } \right)G\left( {\Sigma_{D} - \Sigma_{D}^{\dag } } \right)G^{\dag } } \right]} \right\}} \left[ {f\left( {E - E_{{F_{S} }} } \right) - f\left( {E - E_{{F_{D} }} } \right)} \right]$$

where $Tr$ is trace operator.

## Results and discussion

**Fig. 2:**
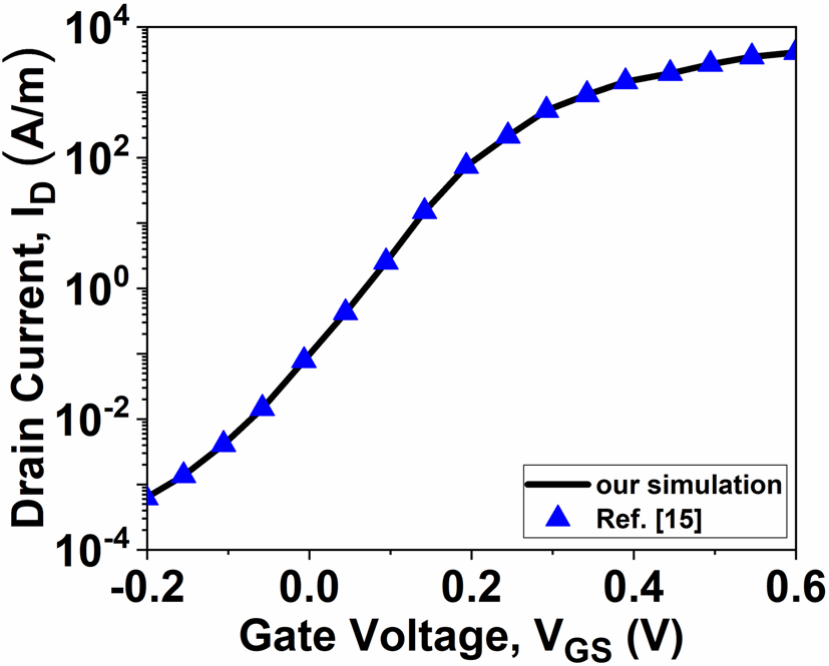
Calibration of the NanoTCAD ViDES tool: Comparison of transfer characteristics with the data reported in^15^.

To ensure the accuracy of the simulations in this study, we first calibrated InSe-FET architectures previously reported in^15^. Figure 2 shows the transfer characteristics, which closely match the results from the earlier study. After calibrating the device simulator, we investigated the effect of oxide materials on device performance. The dielectric materials used in our study, along with their dielectric constants (k), are: *SiO*_2_ (k = 3.9), *Al*_2_*O*_3_ (k = 9), and *HfO*_2_ (k = 21).

**Fig. 3:**
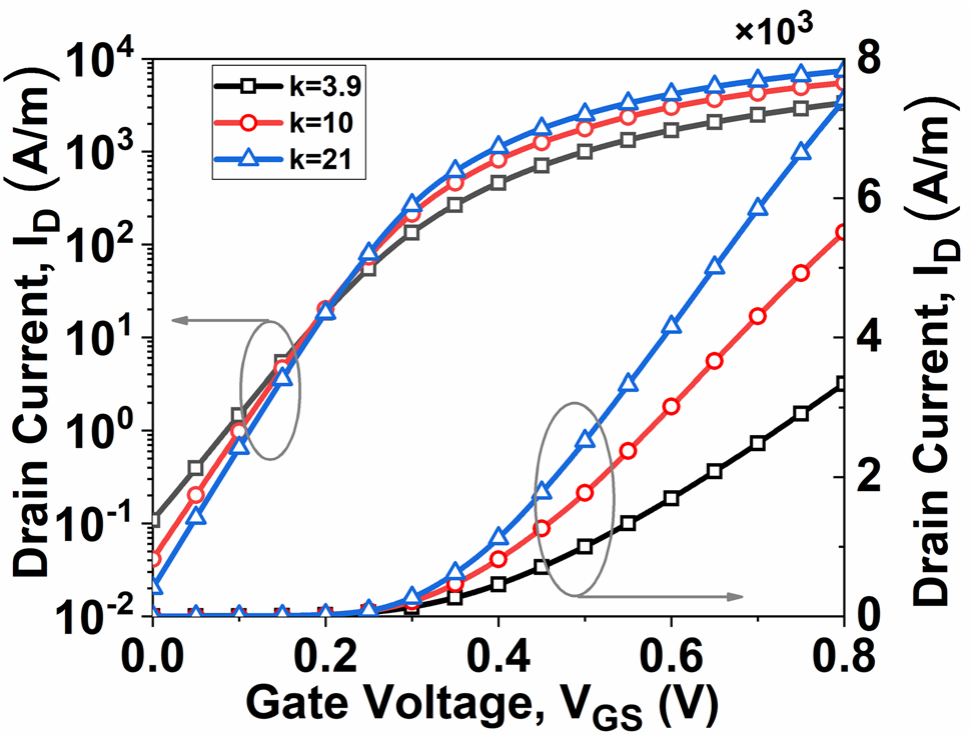
Transfer characteristics of InSe-FETs with different dielectrics at *V*_*DS*_ = 0.5 V.

**Fig. 4:**
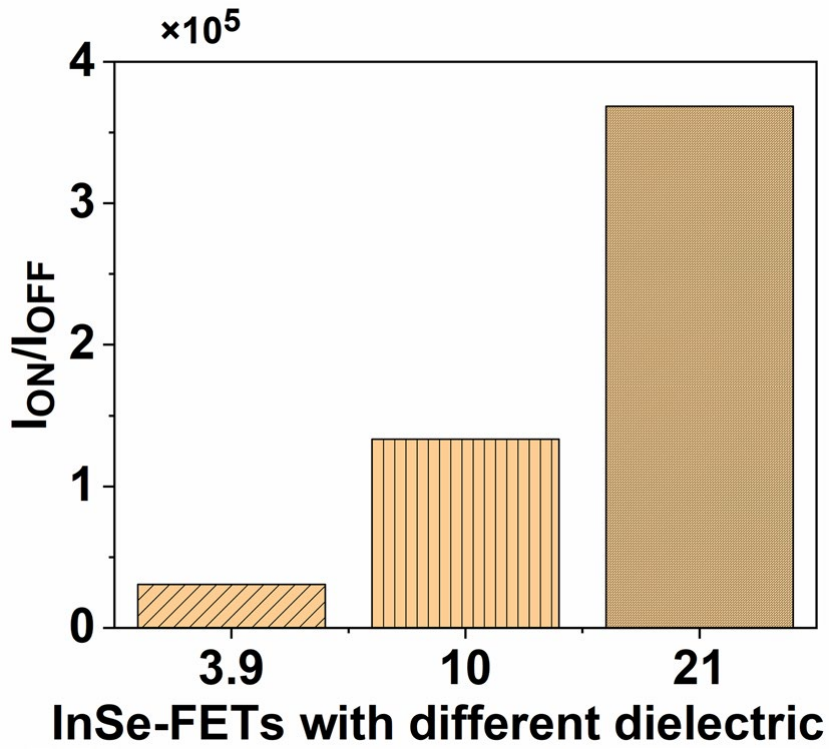
*I*_*ON*_/*I*_*OFF*_ ratio of InSe-FETs with different dielectrics at *V*_*DS*_ = 0.5 V.

The transfer characteristics of InSe-FET, dependent on dielectric strength, are illustrated in Fig. 3. The findings reveal that the ON current is at its minimum for lower dielectric strength and gradually increases with higher dielectric strength, while the variation in OFF current is at its maximum for lower dielectric strength and gradually decreases with higher dielectric strength. Consequently, the *I*_*ON*_/*I*_*OFF*_ ratio exhibits a gradual increase with higher k, as depicted in Fig. 4. Here, *I*_*ON*_ and *I*_*OFF*_ are calculated with a fixed bias voltage *V*_*DD*_ at 0.5 V, as [*I*_*ON*_*, I*_*OFF*_] = [*I*_*DS*_ (*V*_*GS*_ = 0.8 V*, V*_*DS*_ = 0.5 V), *I*_*DS*_ (*V*_*GS*_ = *0* V*, V*_*DS*_ = 0.5 V)].

**Fig. 5:**
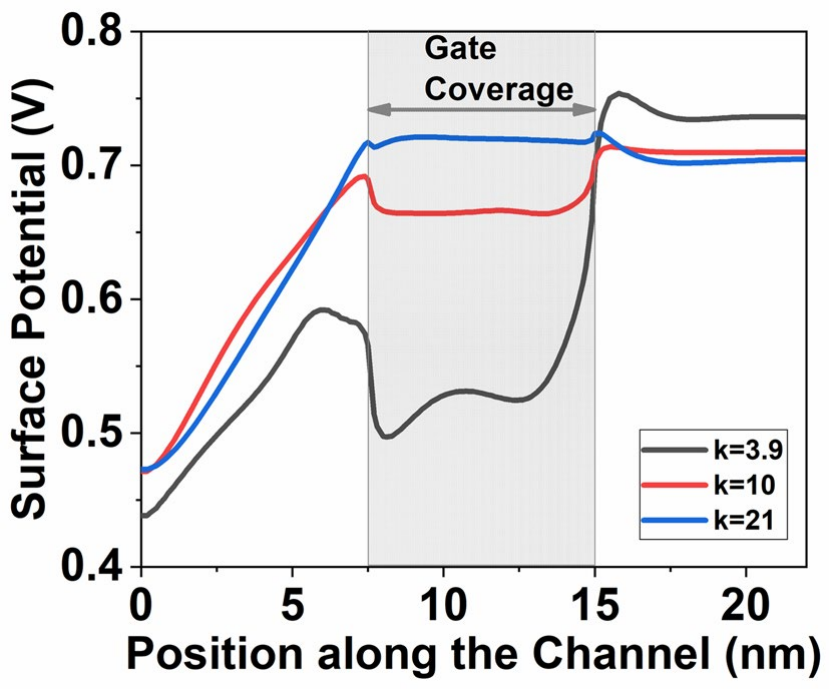
Surface Potential of InSe-FETs with different dielectrics at* V*_*GS*_ = 0.8 V and *V*_*DS*_ = 0.5 V.

The increase in drain current (*I*_*D*_) observed with high-k materials is attributed to gate fringe-induced barrier lowering (GFIBL), as evidenced by the device’s surface potential. Figure 5 illustrates the surface potential of the InSe-FET across the channel for various high-k materials. This figure demonstrates that in an InSe-FET structure, the presence of high-k material induces an inversion charge, thereby reducing the potential barrier. As the dielectric constant increases, both the effective gate capacitance and fringing-field capacitance also increase, which modifies the electrostatics within the channel. This results in a more pronounced fringing-field effect, leading to further reduction of the surface potential barrier. The high-*k* gate dielectric enhances the gate control by increasing the gate capacitance, thereby strengthening the electric field and improving the modulation of charge carriers in the channel. Consequently, the enhanced gate-fringing field facilitates more effective inversion layer formation, which contributes to the observed improvement in current drive capability. Due to the high dielectric constant of the field-effect transistor, the gate-fringing field-induced inversion becomes more pronounced. The high-k dielectric material in the gate helps enhance the capacitance of the gate, leading to a stronger electric field and better control over the flow of charge carriers in the channel. This increased gate-fringing field, in turn, contributes to a more effective induction of inversion in the channel region.

### Analog performance

The simulation and presentation here focus on the influence of high-k material on crucial parameters in analog circuit analysis, such as transconductance (*g*_*m*_), transconductance generation factor (*TGF*), output resistance (*r*_*0*_), output conductance (*g*_*ds*_), intrinsic gain (*A*_*V*_), of the InSe-FET.

The charge-based illustrations of *g*_*m*_ and *g*_*ds*_ are provided by^27^:

7 $$g_{m} = \frac{{\partial I_{D} }}{{\partial V_{GS} }} = (\mu W/L)\cdot(Q_{S} {-}Q_{D} )$$

8 $$g_{ds} = \frac{{\partial I_{D} }}{{\partial V_{DS} }} = \left( {\mu W/L} \right) \cdot Q_{D}$$

9 $$r_{0} = \frac{1}{{g_{ds} }}$$

In the given expressions, *Q*_*S*_ and *Q*_*D*_ denote the charges at the gate-to-source and gate-to-drain ends, respectively. *μ* represents the effective electron mobility, while *W* corresponds to the device’s width.

**Fig. 6:**
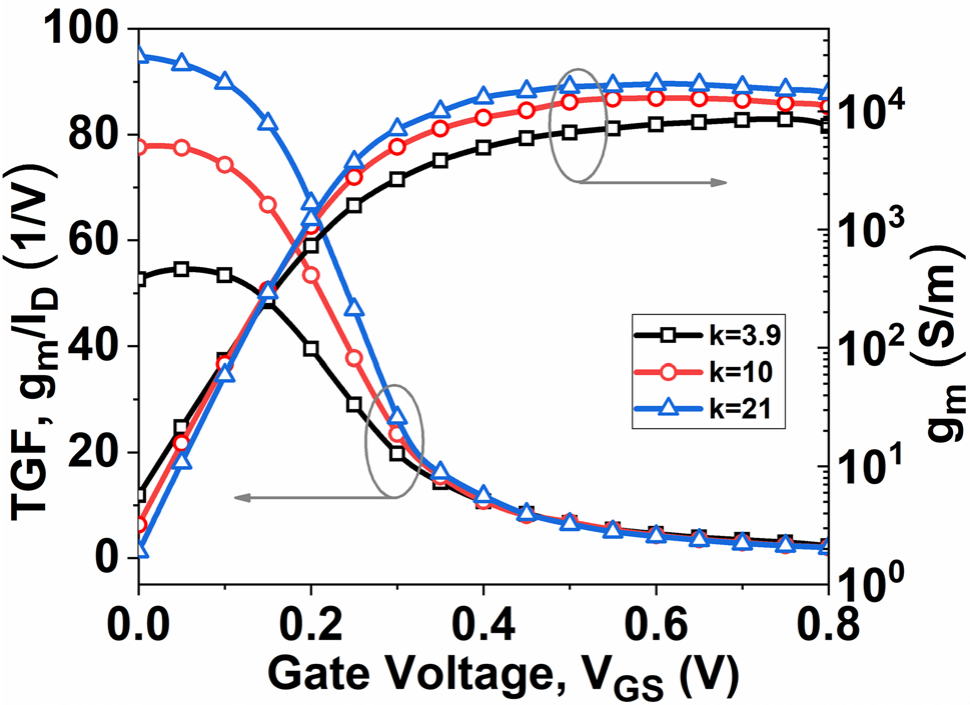
Plot of (**a**) ${g}_{m}$ and TGF with *V*_*GS*_ at *V*_*DS*_ = 0.5 V.

A composite plot illustrating the *g*_*m*_ and TGF (*g*_*m*_*/I*_*D*_) in relation to the gate-to-source voltage (*V*_*GS*_) is depicted in Fig. 6. The *g*_*m*_ of the InSe-FET, crucial for determining the amplifier gain, is expressed in Eq. (7). Figure 6 illustrates that the *g*_*m*_ increases with the increase of high-k material, similar to the drain current. This improvement in transconductance is attributed to enhanced carrier transport efficiency enabled by high-k materials, making them well-suited for analog applications. The increase in TGF is due to the combined effect of higher *g*_*m*_ and lower *I*_*D*_, which arises from an improved drain-to-gate reverse field when high-k materials are used under low gate bias. TGF effectively demonstrates the utilization of current to achieve the desired transconductance value. A high TGF value proves advantageous for realizing analog circuits operating at low supply voltages^28^.

As indicated in Eq. (7), it is observed that the transconductance increases in direct proportion to the difference in source/drain (S/D) charges and the effective electron mobility. In the subthreshold region, the gate control gradually increases in the gate-to-source charge (*Q*_*S*_) for the DG InSe-FET. Simultaneously, the lateral drain field-controlled gate-to-drain charge (*Q*_*D*_) remains nearly constant, given the minimal impact of the gate fringing field. Consequently, the swift increase in *Q*_*S*_ − *Q*_*D*_ results in a rapidly escalating subthreshold transconductance with varying *V*_*GS*_. Under subthreshold bias conditions, the high-k material effect does not lead to a noteworthy enhancement in *Q*_*S*_. Nevertheless, *Q*_*D*_ experiences a slight decrease due to the rise in the reverse drain-to-gate field, resulting in an increased transconductance for a high-k material. In the superthreshold region, where both the gate field and gate fringing field are effective, there is a substantial improvement in both *Q*_*S*_ and *Q*_*D*_. Consequently, the increase in *Q*_*S*_*-Q*_*D*_ is minimal, and *g*_*m*_ remains relatively constant.

**Fig. 7:**
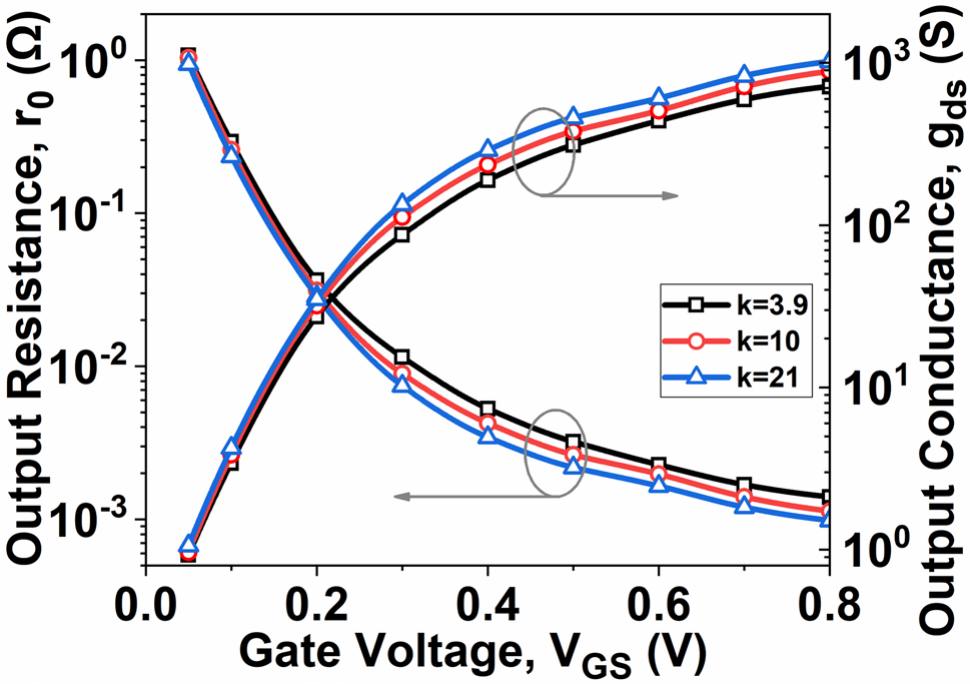
Variation of ${r}_{0}$ and ${g}_{ds}$ as a function of *V*_*GS*_ at *V*_*DS*_ = 0.5 V.

As depicted in Fig. 7, it is noticeable that the device’s *r*_*0*_ reduces with increasing *V*_*GS*_ with high-k material. This behavior is attributed to the higher inversion charge and enhanced gate fringe-induced barrier lowering (GFIBL) associated with high-k materials. Considering Eq. (8), it is evident that *g*_*ds*_ augments with the rise in *Q*_*D*_. Consequently, it can be inferred that the value of *r*_*0*_ derived from 1/*g*_*ds*_ diminishes as *Q*_*D*_ increases. The decrease in *r*_*0*_ due to a high-k material is explained by Eq. (8) and (9), which correlates *g*_*ds*_ with the *Q*_*D*_ controlled by the high-k material. It is noted that an increase in high-k material results in an elevation of the gate fringing field, thereby enhancing *Q*_*D*_ and subsequently increasing *g*_*ds*_. Consequently, the value of *r*_*0*_, represented by 1/*g*_*ds*_, decreases with the high-k. A higher *g*_*ds*_ value indicates an increased efficiency in converting drain current to drain voltage^29^.

Another significant parameter for analog performance is intrinsic gain, which is influenced by the device’s *r*_*0*_ and *g*_*m*_. The determination of intrinsic gain is possible by referring to^30^:

10 $$A_{v} = g_{m} r_{0}$$

**Fig. 8:**
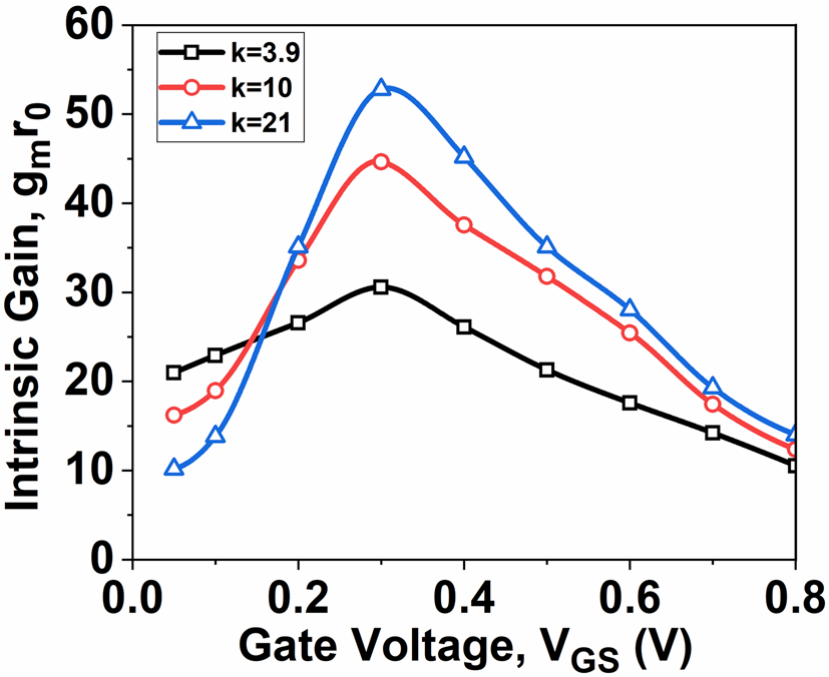
Graph of *g*_*m*_*r*_*0*_ as a function of *V*_*GS*_*.*

Figure 8 illustrates the changes in *g*_*m*_*r*_*0*,_ concerning *V*_*GS*_ at *V*_*DS*_ = 0.5 V. Figure 8 also illustrates that the value of *g*_*m*_*r*_*0*_ rises with high-k material. This increase is primarily attributed to the rise in *g*_*m*_, which increases with the dielectric constant ‘*k*’ and significantly impacts the *g*_*m*_*r*_*0*_ characteristics.

### RF performance

In this section, we analyze the feasibility of utilizing high-k dielectrics in RF applications for devices. We specifically focus on evaluating two critical RF figures of merit (FOMs): gate capacitance (*C*_*G*_) and cut-off frequency (*f*_*T*_).

The significance of *C*_*G*_ as a FOM in RF applications is underscored, and its calculation involves determining the ratio between the change in charge carrier concentration and the change in *V*_*GS*_. The *C*_*G*_ is calculated as follows^31^:

11 $$C_{G} = \frac{dQ}{{dV_{GS} }}$$

**Fig. 9:**
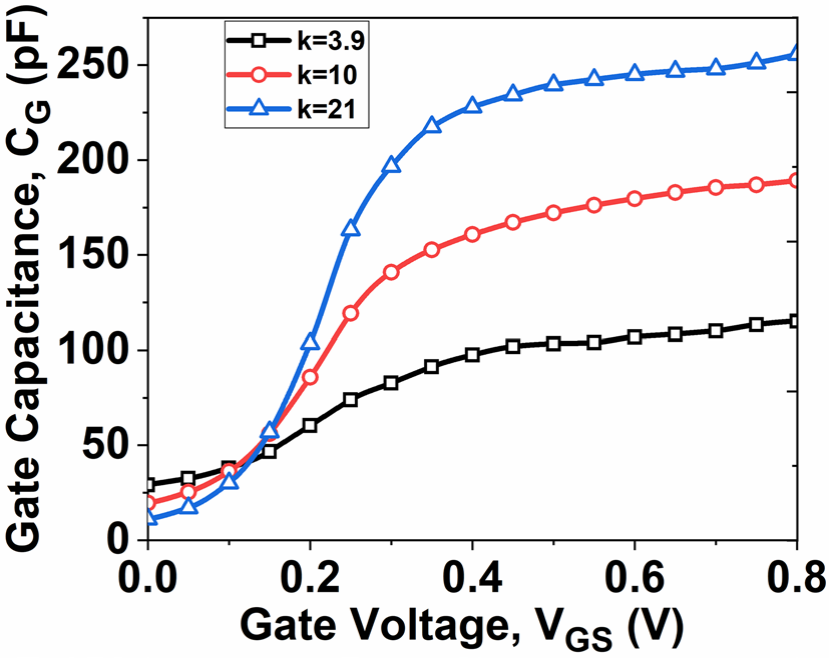
Plot of *C*_*G*_ as a function of *V*_*GS*_*.*

Figure 9 illustrates the gate capacitance of the DG InSe-FET as the high-k dielectric is progressively increased. It is observed that the *C*_*G*_ of the device increases with an increase in the dielectric constant *k*. This increase is due to the higher charge density, resulting from the enhanced fringing field within the high-k material at a given *V*_*GS*_.

A pivotal element in evaluating the RF performance of a device is the cut-off frequency (*f*_*T*_). The frequency at which the current gain equals unity is denoted as *f*_*T*_. The following expression determines the calculation *f*_*T*_:

12 $${f}_{T}={g}_{m}/2\pi {C}_{G}$$

**Fig. 10:**
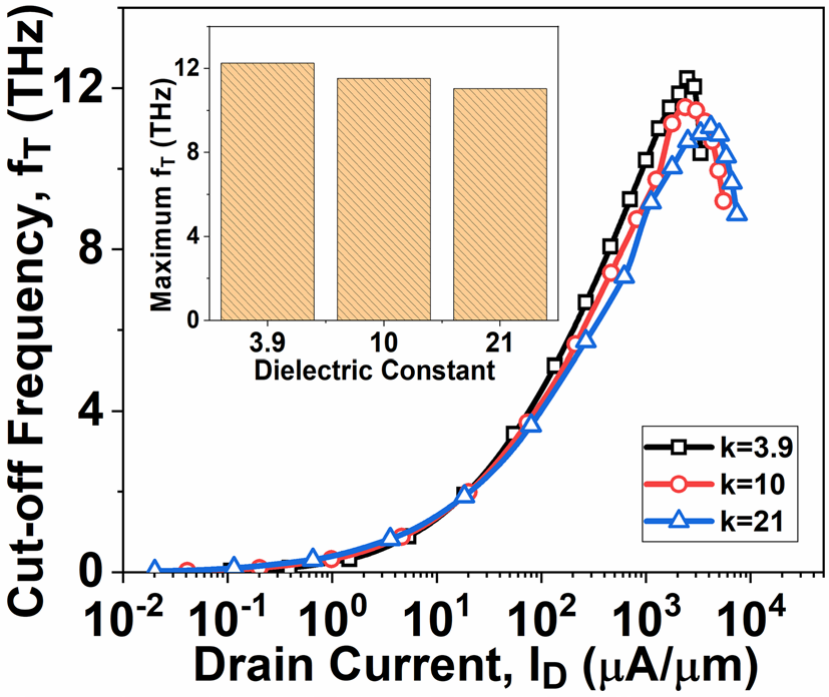
Variation of *f*_*T*_ as a function of *I*_*D*_*.*

Figure 10 shows that the cut-off frequency of the device decreases as the amount of high-k material increases. This reduction in *f*_*T*_ is attributed to changes in the gate capacitance, which significantly affect the frequency characteristics. As illustrated in Fig. 9, the variations in gate capacitance with respect to *V*_*GS*_ are shown. It is observed that gate capacitance increases with rising *V*_*GS*_, especially in devices with high-k dielectrics, due to the enhanced fringing field around the gate sidewalls. Consequently, while the gate capacitance *C*_*G*_ increases, the transconductance of the devices remains relatively stable, as shown in Fig. 6. Consequently, the increased *C*_*G*_ predominantly affects the frequency characteristics of the devices, resulting in decreased *f*_*T*_ for the high-k dielectric device. The inset in Fig. 10 summarizes the maximum *f*_*T*_ values for the device with varying dielectric constant. It highlights that InSe-FETs featuring the device with k values of 25 demonstrate a greater *f*_*T max*_ than those with different dielectric constant values.

After assessing the fundamental RF performance parameters, exploring a critical aspect related to high-speed RF applications is essential. Consequently, finding a balance between device efficiency, intrinsic gain, and bandwidth becomes crucial in analog circuit design. The optimal operating point is determined through trade-off analysis. Consequently, key parameters such as gain frequency product (GFP), transconductance frequency product (TFP), and gain transconductance frequency product (GTFP), are examined.

**Fig. 11:**
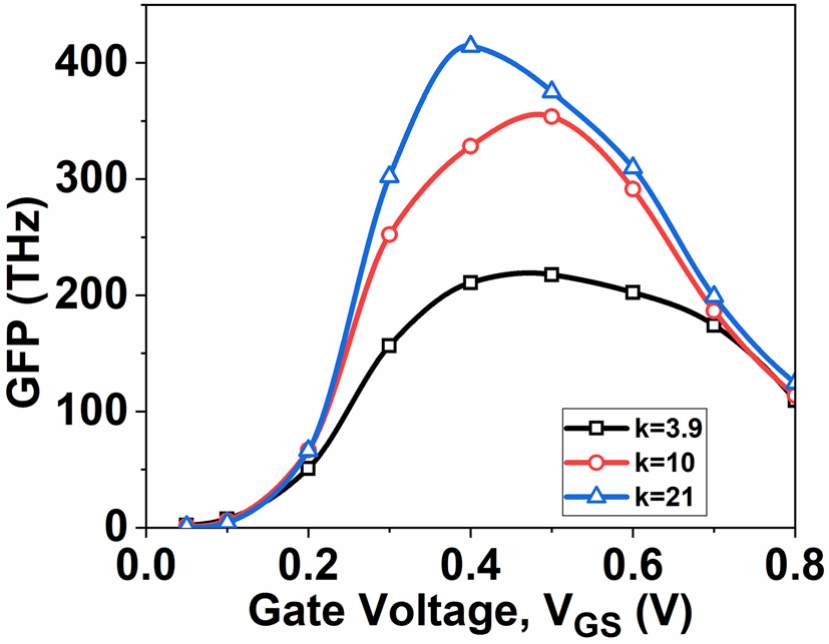
Plot of GFP with *V*_*GS*_ at *V*_*DS*_ = 0.5 V.

The GFP holds significance as a crucial RF parameter, and consequently, the impact of high-k fluctuations on GFP cannot be overlooked. GFP, calculated as the product of gain and frequency, becomes a pivotal factor when employing operational amplifiers in high-frequency applications. In Fig. 11, GFP plot is depicted as a function of *V*_*GS*_. Notably, the GFP value of the device increases with an increase in the dielectric constant k. GFP is particularly relevant for moderate-to-high-speed designs^32^.

**Fig. 12:**
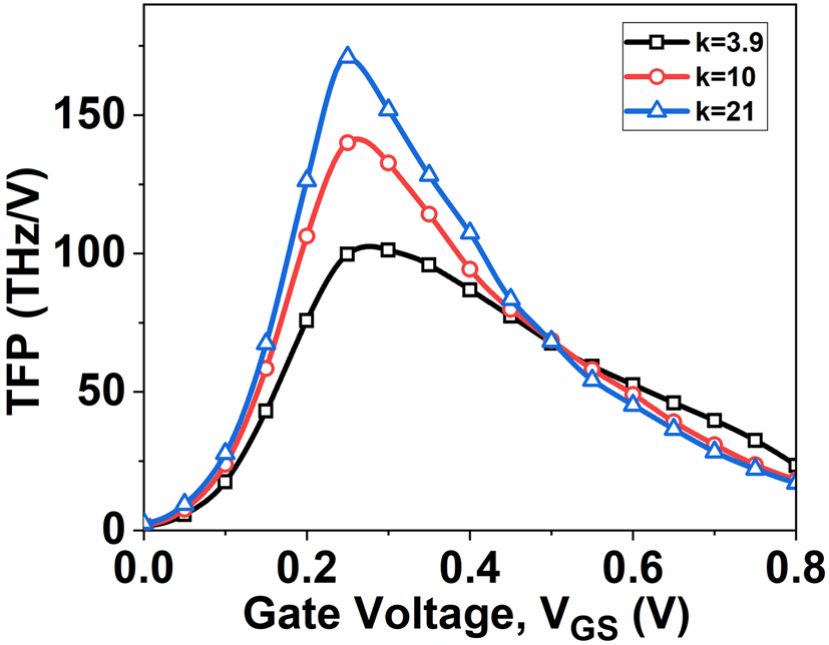
Variation of TFP with *V*_*GS*_ at *V*_*DS*_ = 0.5 V.

TFP, determined as TFP = (*g*_*m*_*/I*_*D*_) *f*_*T*_, is another critical parameter influencing the speed of operation and power consumption. A higher TFP value allows the circuit designer to optimize the operational area by balancing transconductance efficiency and cut-off frequency^33^. Figure 12 displays the TFP graph against *V*_*GS*_, indicating that the TFP value of the device increases with an increase in the dielectric constant k.

**Fig. 13:**
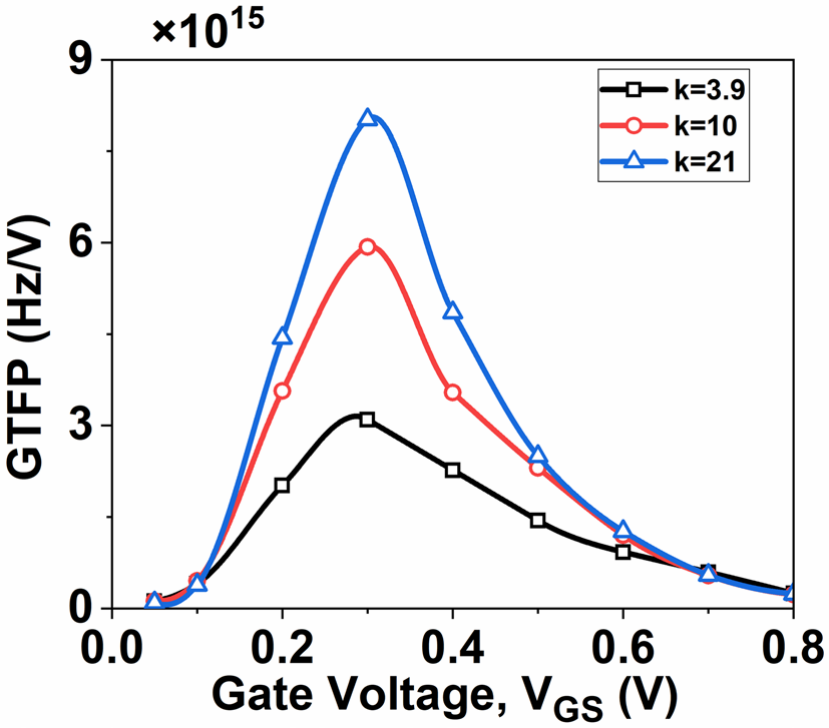
Variation of GTFP with *V*_*GS*_ at *V*_*DS*_ = 0.5 V.

A trade-off analysis considering device efficiency, intrinsic gain, and frequency is essential to identify the optimal operating point in analog circuitry. GTFP, determined as GTFP = (*g*_*m*_*r*_*0*_) (*g*_*m*_*/I*_*D*_)* f*_*T*_, plays a crucial role in this analysis. A higher GTFP value empowers the circuit designer to navigate the best operational area by balancing gain, transconductance efficiency, and cut-off frequency. Figure 13 illustrates the GTFP plot as a function of *V*_*GS*_, showing the GTFP value of the device increases with an increase in the dielectric constant k due to their higher intrinsic gain and enhanced transistor efficiency.

### Performance benchmarking

Table 2 compares the proposed InSe-FET configurations with varying gate dielectric constants (k) against previously reported InSe-FET designs. The data clearly indicate that increasing k enhances both the ON-state current (I_ON_) and the *I*_*ON*_/*I*_*OFF*_ ratio, while also boosting transconductance (g_m_), a key determinant of analog/RF efficiency. For k = 21, the device achieves: 121% improvement in I_ON_, compared to k = 3.9, More than an order is increase in *I*_*ON*_/*I*_*OFF*_ ratio, and nearly two-times enhancement in g_m_. These gains signify improved transistor efficiency, enabling higher gain, faster operation, and superior RF performance at low supply voltages. Consequently, the proposed high-k InSe-FET configuration demonstrates strong potential for next-generation energy-efficient analog and RFIC applications.

**Table 2:** Comparison of proposed InSe-FET with reported devices.

Parameters	Ref.^14^	Ref.^15^	InSe-FET (k = 3.9)	InSe-FET (k = 10)	Proposed InSe-FET (k = 21)
Gate length (nm)	6	7.2	7.5	7.5	7.5
Supply Voltage (V)	0.5	0.5	0.5	0.5	0.5
Dielectric constant	8.6	3.9	3.9	10	21
n-type doping (cm^−2^)	1.5 × ${10}^{13}$	3.6 × 10^13^	3.6 × 10^13^	3.6 × 10^13^	3.6 × 10^13^
Oxide thickness (nm)	0.5	0.5	0.5	0.5	0.5
I_ON_ (A/m)	2.5 × ${10}^{3}$	–	3.34 × ${10}^{3}$	5.5 × ${10}^{3}$	7.4 × ${10}^{3}$
*I*ON/*I*OFF	${10}^{4}$	2.7 × ${10}^{4}$	3.07 × ${10}^{4}$	1.33 × ${10}^{5}$	3.68 × ${10}^{5}$
*g*_*m*_ (S/m)	13 × ${10}^{3}$	–	8.6 × ${10}^{3}$	13 × ${10}^{3}$	17 × ${10}^{3}$

## Conclusion

This paper presents a systematic investigation into the performance of a DG InSe-FET incorporating high-k dielectric materials. It demonstrates how high-k dielectric devices enhance the performance of the DG InSe-FET across various analog and RF parameters. Furthermore, it explores the impact of high-k dielectric on some derived RF performance parameters, such as GFP, TFP, and GTFP, which are critical parameters for analog circuit design. The analysis reveals that employing higher dielectric constants enhances GFP and GTFP due to increased intrinsic gain, whereas TFP improves due to the enhancement of transistor efficiency, TGF. The GFP, GTFP, and TFP show approximately 90.4%, 159%, and 68.7% improvements, respectively, with a high-k value of 21 compared to 3.9. However, the device’s cut-off frequency experiences a notable decline of 9.85% with the high-k device. Therefore, careful optimization of the dielectric constant is essential depending on whether the target application prioritizes intrinsic gain, cut-off frequency, or RF performance. As a future extension of this work, we plan to incorporate non-ideal effects such as scattering and defects into the simulation framework to achieve more realistic modeling aligned with experimental observations.

## Supplementary Information

Supplementary Information.

## Data Availability

The datasets used and/or analyzed during the current study available from the corresponding author, [bhubon1126@gmail.com], or from the first author, [akram14407@gmail.com], on reasonable request.

## References

[1] Duan, X., Wang, C., Pan, A., Yu, R. & Duan, X. Two-dimensional transition metal dichalcogenides as atomically thin semiconductors: Opportunities and challenges. *Chem. Soc. Rev.* **44**(24), 8859–8876. 10.1039/c5cs00507h (2015). 26479493 10.1039/c5cs00507h

[2] Jena, D., Banerjee, K. & Xing, H. G. 2D crystal semiconductors: Intimate contacts. *Nat. Mater.* **13**, 1076–1078. 10.1038/nmat4121 (2014). 25410976 10.1038/nmat4121

[3] Huang, W., Gan, L., Li, H., Ma, Y. & Zhai, T. 2D layered group IIIA metal chalcogenides: Synthesis, properties and applications in electronics and optoelectronics. *CrystEngComm* **8**(22), 3968–3984. 10.1039/C5CE01986A (2016).

[4] Bandurin, D. A. et al. High electron mobility, quantum Hall effect and anomalous optical response in atomically thin InSe. *Nat. Nanotechnol.* **12**, 223–227. 10.1038/nnano.2016.242 (2016). 27870843 10.1038/nnano.2016.242

[5] Sucharitakul, S. et al. Intrinsic electron mobility exceeding 10^3^ cm^2^/(V s) in multilayer InSe FETs. *Nano Lett.* **15**(6), 3815–3819. 10.1021/acs.nanolett.5b00493 (2015). 25924062 10.1021/acs.nanolett.5b00493

[6] Lei, S. et al. Evolution of the electronic band structure and efficient photo-detection in atomic layers of InSe. *ACS Nano* **8**(2), 1263–1272. 10.1021/nn405036u (2014). 24392873 10.1021/nn405036u

[7] Balakrishnan, N. et al. Room temperature electroluminescence from mechanically formed van der Waals III–VI homojunctions and heterojunctions. *Adv. Opt. Mater.* **2**(11), 1064–1069. 10.1002/adom.201400202 (2014).

[8] Ho, C.-H. & Chu, Y.-J. Bending photoluminescence and surface photovoltaic effect on multilayer InSe 2D microplate crystals. *Adv. Opt. Mater.* **3**(12), 1750–1758. 10.1002/adom.201500390 (2015).

[9] Tamalampudi, S. R. et al. High performance and bendable few-layered InSe photodetectors with broad spectral response. *Nano Lett.* **14**(5), 2800–2806. 10.1021/nl500817g (2014). 24742243 10.1021/nl500817g

[10] Lei, S. et al. An atomically layered InSe avalanche photodetector. *Nano Lett.* **15**(5), 3048–3055. 10.1021/acs.nanolett.5b00016 (2015). 25822539 10.1021/acs.nanolett.5b00016

[11] Chen, Z., Biscaras, J. & Shukla, A. A high performance graphene/few-layer InSe photo-detector. *Nanoscale* **7**(14), 5981. 10.1039/C5NR00400D (2015). 25775954 10.1039/c5nr00400d

[12] Chang, P., Liu, X., Liu, F. & Du, G. Phonon-limited mobility in n-type few-layer InSe devices from first principles. *IEEE Electron Device Lett.* **40**(2), 333–336. 10.1109/LED.2018.2886842 (2019).

[13] Chang, P., Liu, X., Liu, F. & Du, G. Remote phonon scattering in two-dimensional InSe FETs with High-κ gate stack. *Micromachines* **9**(12), 674. 10.3390/mi9120674 (2018). 30572574 10.3390/mi9120674 PMC6316064

[14] Ahn, Y. & Shin, M. First-principles-based quantum transport simulations of monolayer indium selenide FETs in the ballistic limit. *IEEE Trans. Electron Devices* **64**(5), 2129–2134. 10.1109/TED.2017.2679217 (2017).

[15] Marin, E. G., Marian, D., Iannaccone, G. & Fiori, G. First-principles simulations of FETs based on two-dimensional InSe. *IEEE Electron Device Lett.* **39**(4), 626–629. 10.1109/LED.2018.2804388 (2018).

[16] Chang, P., Liu, X., Liu, F. & Du, G. First-principles based ballistic transport simulation of monolayer and few-layer InSe FETs. *Japan. J. Appl. Phys.* **58**(SB), SBBA02. 10.7567/1347-4065/aafb4f (2019).

[17] Feng, W., Zheng, W., Cao, W. & Hu, P. Back gated multilayer InSe transistors with enhanced carrier mobilities via the suppression of carrier scattering from a dielectric interface. *Adv. Mater.* **26**(38), 6587–6593. 10.1002/adma.201402427 (2014). 25167845 10.1002/adma.201402427

[18] Sucharitakul, S. et al. Intrinsic electron mobility exceeding 10^3^ cm^2^/(Vs) in multilayer InSe FETs. *Nano Lett.* **15**(6), 3815–3819. 10.1021/acs.nanolett.5b00493 (2015). 25924062 10.1021/acs.nanolett.5b00493

[19] International Roadmap for Devices and Systems, “More moore”, https://irds.ieee.org/editions/2021/more-moore, 2021.

[20] G. Fiori and G. Iannaccone, NanoTCAD ViDES, accessed on [Online] Available: http://vides.nanotcad.com/vides/ (2024)

[21] Lovarelli, G., Calogero, G., Fiori, G. & Iannaccone, G. Multiscale pseudoatomistic quantum transport modeling for van der Waals heterostructures. *Phys. Rev. Appl.* **18**, 034045. 10.1103/PhysRevApplied.18.034045 (2022).

[22] M. Aliofkhazraei, et al., “Graphene Science Handbook Nanostructure and Atomic Arrangement,” *CRC Press Inc*., May 2016, ISBN: ISBN 9781466591370 - CAT# K20510.

[23] Datta, S. Nanoscale device modeling: The Green’s function method. *Superlattice Microstruct.* **28**, 253–278. 10.1006/spmi.2000.0920 (2000).

[24] http://www.gianlucafiori.org/articles/ViDESmanual.pdf

[25] Datta, S. *Quantum Transport: Atom to Transistor* (Cambridge University Press, 2013).

[26] Trellakis, A., Galick, A. T., Pacelli, A. & Ravaioli, U. Iteration scheme for the solution of the two-dimensional Schrodinger-Poisson equations in quantum structures. *J. Appl. Phys.* **81**(12), 7880–7884. 10.1063/1.365396 (1997).

[27] Moldovan, O. et al. Explicit analytical charge and capacitance models of undoped double-gate MOSFETs. *IEEE Trans. Electron Devices* **54**(7), 1718–1724. 10.1109/TED.2007.899402 (2007).

[28] Tripathy, M. R. et al. Device and circuit-level assessment of GaSb/Si heterojunction vertical tunnel-FET for low-power applications. *IEEE Trans. Electron Devices* **67**(3), 1285–1292. 10.1109/TED.2020.2964428 (2020).

[29] Ahmad, M. A. et al. Trade-off analysis between gm/ID and fT of GNR-FETs with single-gate and double-gate device structure. *Sci. Rep.* **14**, 10218. 10.1038/s41598-024-59908-5 (2024). 38702353 10.1038/s41598-024-59908-5 PMC11068727

[30] Ahmad, M. A. & Kumar, J. The understanding of the impact of efficiently optimized underlap length on analog/RF performance parameters of GNR-FETs. *Sci. Rep.* **13**, 13872. 10.1038/s41598-023-40711-7 (2023). 37620403 10.1038/s41598-023-40711-7 PMC10449920

[31] Hiblot, G., Rafhay, Q., Boeuf, F. & Ghibaudo, G. Analytical model for the inversion gate capacitance of DG and UTBB MOSFETs at the quantum capacitance limit. *IEEE Trans. Electron Devices* **62**(5), 1375–1382. 10.1109/TED.2015.2406116 (2015).

[32] Mohapatra, S. K. et al. Estimation of analog/RF figures-of-merit using device desgn engineering in gate stack double gate MOSFET. *Mater. Sci. Semicond. Process.* **31**, 455–462. 10.1016/j.mssp.2014.12.026 (2015).

[33] Ahmad, M. A., Maurya, A. & Kumar, J. Investigation of pocket engineering in GNR tunnel-FET using Analog/RF figures of merit. *IEEE Trans. Electron Devices* **71**(12), 7921–7927. 10.1109/TED.2024.3484349 (2024).

