# Supplementary material for: Performance optimization of InSe-FETs using high-k dielectric materials for analog/RF applications
Source: Sci Rep. 2026 Mar 10;16:9573. doi: 10.1038/s41598-025-21242-9 (PMC13009511; doi:10.1038/s41598-025-21242-9)
Supplement: Supplementary file 1 — Supplementary Information. [file 41598_2025_21242_MOESM1_ESM.docx]

**Appendix A**

***Gate Charge Calculation Methodology:***

In the NEGF framework, the NEGF framework, terminal charges are obtained from the spatial charge distribution along the device channel. The procedure for computing the total gate charge involves three main steps: (i) extracting the electron and hole density profiles along the channel from the NEGF formalism [24], (ii) converting the summed electron density into charge using the elementary charge q, and (iii) integrating the resulting charge density over the gate-controlled region to obtain the total gate–channel charge (Q).

Finally, the gate capacitance is calculated using [31]:

$C_{G}$ = $\frac{dQ}{dV_{GS}}$ .

**Reference**

[24] <http://www.gianlucafiori.org/articles/ViDESmanual.pdf>

[31] G. Hiblot, Q. Rafhay, F. Boeuf and G. Ghibaudo, "Analytical Model for the Inversion Gate Capacitance of DG and UTBB MOSFETs at the Quantum Capacitance Limit," *IEEE Transactions on Electron Devices*, vol. 62, no. 5, pp. 1375-1382, May 2015, doi: 10.1109/TED.2015.2406116.
